# An Analysis Method for Capacitive Micromachined Ultrasound Transducer (CMUT) Energy Conversion during Large Signal Operation

**DOI:** 10.3390/s19040876

**Published:** 2019-02-20

**Authors:** Amirabbas Pirouz, F. Levent Degertekin

**Affiliations:** 1 School of Electrical and Computer Engineering, Georgia Institute of Technology, Atlanta, GA 30332, USA; levent.degertekin@me.gatech.edu; 2 Woodruff School of Mechanical Engineering, Georgia Institute of Technology, Atlanta, GA 30332, USA

**Keywords:** CMUT coupling coefficient, CMUT efficiency, CMUT energy analysis, CMUT large signal operation

## Abstract

With Capacitive Micromachined Ultrasound Transducers (CMUTs) increasingly being used for high intensity, large signal ultrasound applications and several drive methods being proposed, the efficiency of these devices in this operation regime have not been quantitatively evaluated. Since well-known frequency and capacitance-based coupling coefficients definitions are not valid for large signal, nonlinear operation, an energy-based definition should be used. In this paper, an expression for mechanical energy in a CMUT is obtained based on the assumption that CMUT is a linear time varying capacitor in all regimes of operation. This expression is evaluated by the help of an experimentally verified nonlinear CMUT model to define an energy conversion ratio (ECR) which can be considered as a coupling coefficient valid for all regimes of operation. This parameter is validated in the small signal regime and then used to evaluate CMUT performance with various large drive signals. The quantitative modeling results show that CMUTs do not need DC bias to achieve high efficiency large signal transduction: AC only signals at half the operation frequency with amplitudes beyond the collapse voltage can provide efficiencies (ECR) above 0.9 with harmonic content below −25 dB. Based on these results, ECR variation with membrane geometry and parasitic capacitance are given as examples for device optimization. The overall modeling approach is also qualitatively validated by experiments.

## 1. Introduction

Electromechanical coupling coefficients are frequently used to evaluate transducer performance [1]. The definitions used to calculate and measure this coefficient for CMUTs follow the usual small signal approximation which assumes that the energy conversion occurs at the input frequency and the coupling coefficient is independent of the drive level [1,2,3]. With these constraints, the coupling coefficient for a CMUT can be obtained by capacitance and resonance frequency measurements, similar to piezoelectric transducers [4,5].

In many applications however, the CMUTs are operated with large input signals to increase the output pressure. These include pulsed operation where the input pulse voltage is beyond the collapse voltage of the CMUT [6], and more importantly continuous wave operation for applications like high intensity focused ultrasound (HIFU) [7,8,9,10]. Furthermore, CMUTs can be used with different large signal CMUT drive schemes for high power applications, such as no DC bias operation [7,10,11]. In these applications, linear CMUT models and small signal approximations are not valid. These previous studies involved device and drive signal optimization, but the approach was mainly experimental and efficiency of operation was not explored. In order to fairly evaluate and compare CMUT performance in these large signal drive schemes, nonlinear transducer models and energy based coupling coefficient definitions, or energy conversion metrics are needed. These metrics, once well-defined, can also be used to optimize CMUT design and operation mode for any large signal and high power applications. For example, the membrane geometry can be adjusted to have higher conversion efficiency, as shown earlier for coupling coefficient of CMUTs [12]. The aim of this paper is to define a model based performance metric for large signal CMUT operation, called energy conversion ratio (ECR), and use it to analyze and optimize CMUT performance. ECR uses the energy based coupling coefficient approach in contrast to the capacitance and resonance frequency measurements, which are only valid for small signal operation (Table 1). The electrical and mechanical energy quantities are obtained using an experimentally verified large signal CMUT model [13] so that the results can be used for all input signal levels.

In this paper, we briefly discuss the large signal CMUT model [13] and describe how we use this model to evaluate a simple expression to determine the mechanical energy stored in the CMUT, both in small and large signal operation regime. Small signal coupling coefficient based on ECR is then verified by comparison with the usual definitions and the need for ECR is demonstrated by comparing linear coupling coefficient with ECR as a function of normalized AC drive level. We then use ECR to analyze and compare large signal CMUT operation with DC biased and AC only operation. We further employ ECR to assess the large signal efficiency of a CMUT with uniform and non-uniform membrane to demonstrate optimization capability. The validity of the mechanical energy predictions are also tested experimentally through output pressure spectrum and intensity measurements.

## 2. CMUT Operation and Nonlinear Model

**Figure 1:**
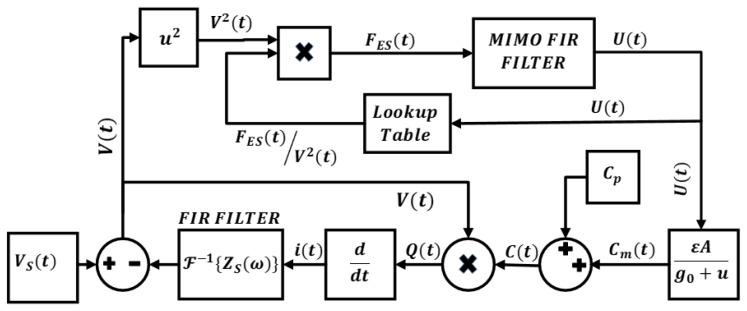
The Simulink model of the CMUT and calculation method.

In order to analyze large signal operation of CMUT, a model which takes into account the nonlinear electrostatic forces with large membrane displacements is employed while the acoustic interactions are modeled in linear regime [13]. In the transmit system Simulink model shown in Figure 1, the CMUT is considered as linear time-varying capacitor, $C\left(t\right)=C_{m}\left(t\right)+C_{P}$, where $C_{m}\left(t\right)$ is the moving time varying part of the capacitor and $C_{P}$ is the static, non-moving part of the capacitance, i.e., parasitic capacitance. Transmit circuitry is modeled with the source impedance ($Z_{s}$) assuming the interface circuit is linear within the operation range [14].

Given the source voltage $v_{s}$(t), which includes both DC and AC components, the instantaneous voltage, v(t) and current, i(t) on the CMUT is given as in Equation (1):

(1) $$\begin{array}{r} v\left(t\right)=v_{S}\left(t\right)-i\left(t\right)*{ℱ}^{-1}\left\{Z_{S}\left(\omega \right)\right\}\\ i\left(t\right)=\frac{dQ\left(t\right)}{dt}=\frac{dv\left(t\right)\left(C_{m}\left(t\right)+C_{p}\right)}{dt} \end{array}$$

where ${ℱ}^{-1}\left\{Z_{S}\left(\omega \right)\right\}$ is the inverse Fourier transform of the source impedance, Q(t) is the charge on the CMUT capacitance C(t). The impact of higher order membrane modes are included by dividing the moving electrode into patches with related electrostatic force distribution. The total capacitance is found by adding parallel plate approximated capacitance from each patch, $C_{i}\left(t\right)$:

(2) $$C\left(t\right)=\underset{i}{\sum }C_{mi}\left(t\right)+C_{p}=\underset{i}{\sum }\frac{\varepsilon_{o}Ai}{g_{0}+u_{i}\left(t\right)}+C_{p}$$

Here the *A_i_* is the area of the *i*th patch, $\varepsilon_{o}$ is the permititivity, $g_{0}$ is the initial effective gap and $u_{i}\left(t\right)$ is the displacement of the *i*th patch. To ensure the CMUT operates in non-collapse mode, membrane displacements are limited by the gap height which controlled by a conditional command. The multi input multi output (MIMO) finite impulse response (FIR) filter block in Simulink model describes the acoustic response of each patch in accordance with the electrostatic force ($F_{ES}$) and patch displacement vectors (U(t)). The surface of the CMUT is meshed by the boundary element method (BEM) and this MIMO FIR block is derived by solving the system of force equations G(ω) on each node [13]. G(ω) is the matrix that relates stiffness (K), mass (M) and fluid coupling ($Z_{r}\left(\omega \right)$) matrices given by Equation (3):

(3) $$G\left(\omega \right)=K-\omega^{2}M+j\omega \left(Z_{r}\left(\omega \right)+b\right)$$

In order to model the mechanical loss in the CMUT membrane structure, the damping proportional to the velocity, b, is added to fluid coupling. Since the electrical source is the only source of energy input, given i(t) and v(t), the total instantaneous power input to the CMUT, p(t), can be calculated by:

(4) p(t)=i(t)v(t)

This expression can be integrated to find the total electrical input energy ($E_{total}$) that is defined as:

(5) $$E_{total}=\int p\left(t\right)dt=\int i\left(t\right)v\left(t\right)dt$$

This energy level is the total input energy used in the energy definition of coupling coefficient shown in the last row of Table 1, as the electrical source is the only energy source.

## 3. ECR Calculation

The critical information required for energy based coupling coefficient calculation for large signal operation is the mechanical energy or work done by the input electrical energy. This is obtained by considering the CMUT as a linear time-varying capacitor, i.e. q(t)=C(t)v(t), and separating the instantaneous power input to the system into two components. The circuit delivers energy to the CMUT capacitor at the rate of p(t) as in Equation (4). While some of this energy is stored as electrostatic energy $E_{c}\left(t\right)$ in the capacitance, the remainder of the energy is the mechanical work done [15]. This is clearly seen in the following Equation (6):

(6) $$\begin{array}{c} p\left(t\right)=i\left(t\right)v\left(t\right)=\frac{dq}{dt}\;v\left(t\right)\;\overset{q\left(t\right)=C\left(t\right)v\left(t\right)}{\Rightarrow }\\ =\frac{d\left(C\left(t\right)v\left(t\right)\right)}{dt}v\left(t\right)=\dot{C}\left(t\right)v{\left(t\right)}^{2}+C\left(t\right)\dot{v}\left(t\right)v\left(t\right)\\ =\frac{1}{2}\dot{C}\left(t\right)v{\left(t\right)}^{2}+\left(\frac{1}{2}\dot{C\left(t\right)}v{\left(t\right)}^{2}+C\left(t\right)\dot{v}\left(t\right)v\left(t\right)\right)\\ =\frac{1}{2}\dot{C}\left(t\right)v{\left(t\right)}^{2}+\frac{d}{dt}\left(\frac{1}{2}C\left(t\right)v{\left(t\right)}^{2}\right)\\ =\frac{1}{2}\dot{C}\left(t\right)v{\left(t\right)}^{2}+\frac{dE_{c}}{dt} \end{array}$$

We assert the difference between p(t) and $\frac{dE_{c}}{dt}$, ($\frac{1}{2}\dot{C}\left(t\right)v{\left(t\right)}^{2}$), $\dot{C}\left(t\right)$ denoting the time derivative of capacitance, is the instantaneous mechanical power input to the CMUT by the electrostatic forces in the capacitor. It is interesting to note that the unit of $\dot{C}\left(t\right)$ is mho, conductance unit which models the mechanical power as power lost in a resistor. By integrating this quantity, the mechanical energy in the general energy based definition of coupling coefficient can be obtained for the CMUT. We denote this ratio as Energy Conversion Ratio (ECR) (Equation (7)) to prevent confusion with the regular coupling coefficient, which is valid only for linear small signal case:

(7) $$ECR=\frac{Mechanical\;Energy}{\left(Mechanical+Electrical\right)\;Energy}=\frac{\int \frac{1}{2}\dot{c}\left(t\right)v{\left(t\right)}^{2}dt}{\int i\left(t\right)v\left(t\right)dt}$$

In addition to handling large signal operation, ECR can be calculated for different membrane geometries for design optimization as the acoustic model of Equation (3) allows for arbitrary *K* and *M* matrices [13]. Similarly, impact of parasitic capacitance $C_{p}$ is inherently included in the total capacitance as in Equation (2).

### ECR Calculation Example

**Figure 2:**
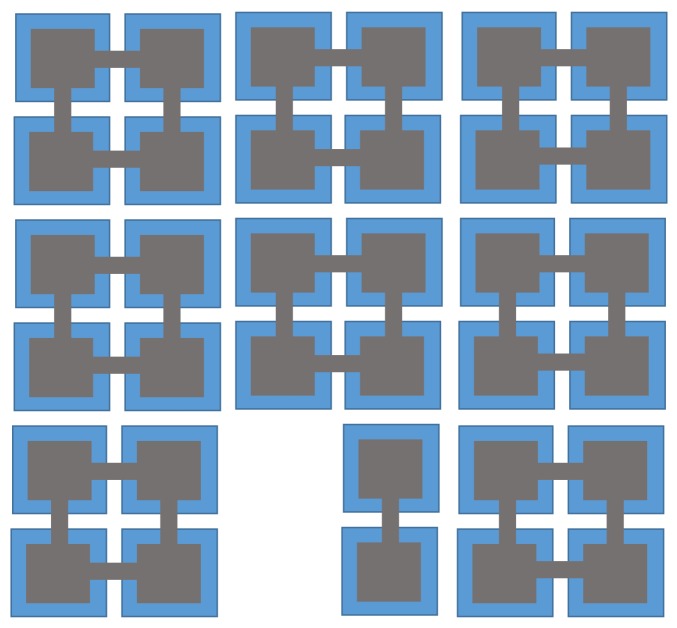
The array used in the simulation and discussed in Table 2. The calculations are for the 4 center membranes and adjacent membranes include array behavior including crosstalk in the simulation.

In order to observe how the CMUT variables related to ECR and ECR itself change over time during dynamic operation, an example calculation is performed on a CMUT with properties listed in Table 2 and geometry shown in Figure 2. This CMUT was designed for an intracardiac imaging application and is used here as a representative CMUT sample. 

**Figure 3:**
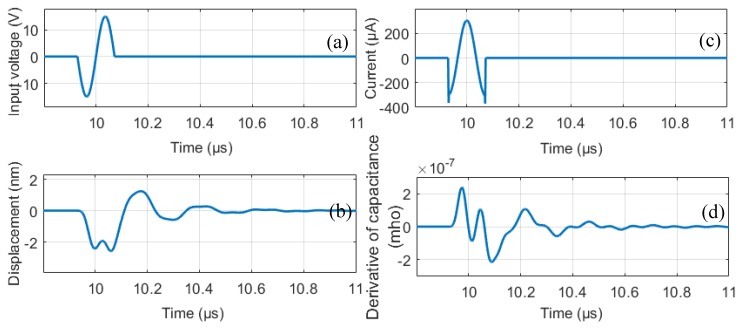
(**a**) The input voltage over the CMUT. (**b**) The membrane average displacement profile. (**c**) The current flow on the CMUT. (**d**) Derivative of the capacitance, which is used in the mechanical energy calculation.

Figure 3 shows the voltage, the average displacement of a CMUT membrane, corresponding current and the time derivative of total capacitance for this single membrane. With these variables, one can calculate the instantaneous power input to the CMUT and the mechanical power dissipated, based on Equations (4) and (6), respectively. Note that, for this particular case, no DC bias is applied to the CMUT membrane.

**Figure 4:**
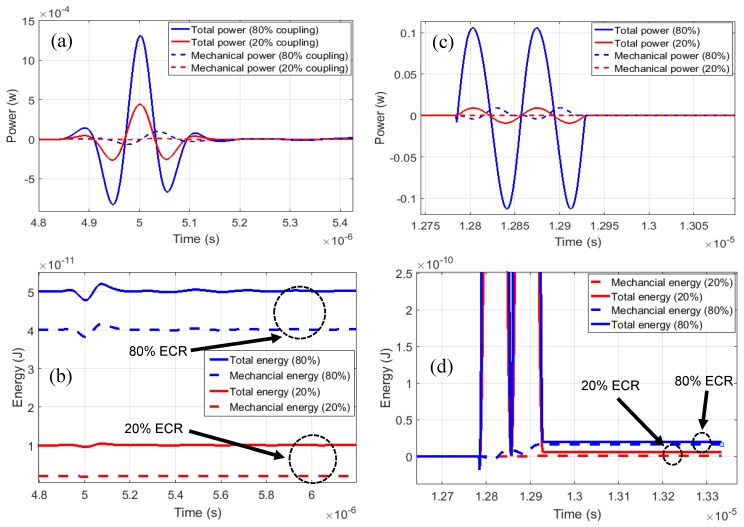
Small signal (**a**) mechanical and total power and (**b**) energy (power integral) variation over time. For the calculation, the DC level is 0.62 × Vcol and 0.95 × Vcol for 20% and 80% coupling, respectively and 0.1 V Gaussian pulse is applied. (**c**) Large signal mechanical and total power and (**d**) energy calculation (power integral) in AC only operation.

The energy variables for ECR are then calculated by integrating the instantaneous power, which can be performed both for transient pulsed operation as well as long tone burst, or close to CW operation, for any DC and AC bias combinations. Figure 4 shows a set of calculations in small signal regime for low and high coupling conditions, which depends on the applied DC bias. In Figure 4a, the solid lines indicate the instantaneous input power whereas the dashed lines show the mechanical power in the CMUT for 20% (red) and 80% (blue) ECR. Note that, although the peak values of the input to mechanical power ratio seem very different, when the energy is calculated through integration to obtain ECR, it is seen that these ratios are indeed 80% and 20%. Also note that, with DC bias and small signal operation the ECR values are independent of time since the coupling coefficient is determined quasi-statically by the DC bias. Figure 4c shows the same calculation as Figure 4a but this time the CMUT is operated without DC bias by applying a large AC only signal. In this case, ECR is zero before the application of the input signal since there is no energy (stored or dynamically changing) on the CMUT before the input signal. However, ECR increases to 80% (blue) or 20% (red) depending on the input level, i.e. higher input level (blue) resulting in higher ECR. This is different from the small signal case, where ECR or coupling coefficient is determined by the DC bias but not by the input level. Therefore, this example clearly illustrates the added capability of handling large signal operation of CMUTs with the proposed method.

**Figure 5:**
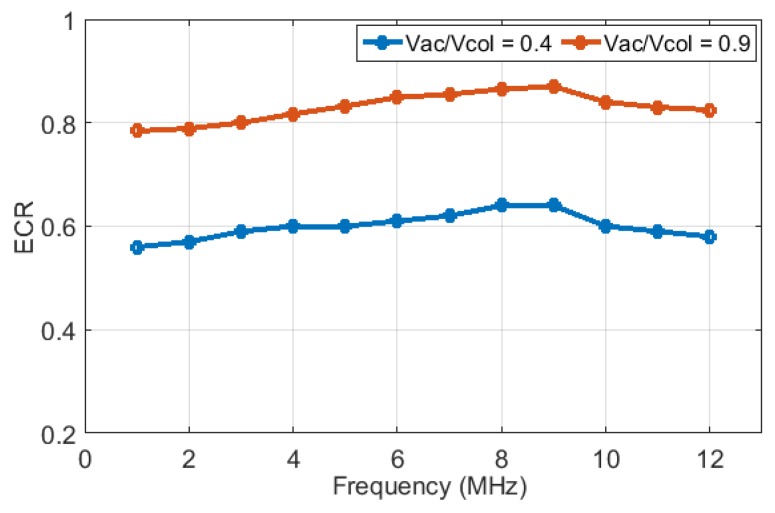
ECR shows relatively similar values in different frequencies where Vac/Vcol is set to 0.4 and 0.9.

Note that unlike small signal coupling coefficient which can be obtained assuming quasi-static operation [1], ECR is obtained through dynamic variables. Therefore, its frequency dependence needs to be considered. This frequency dependence is explored by applying 100-cycle tone burst signals calculating ECR in the 1–12 MHz range for the example CMUT with 9 MHz center frequency in immersion. Figure 5 shows the variation of ECR with frequency for large AC only signals for two cases, where Vac = 0.4 × Vcol and Vac = 0.9 × Vcol.

As explained in the Appendix A, for AC-only excitation cases a voltage signal at the half of the desired output frequency is applied to the CMUT. As expected, ECR values for higher AC signal case are higher in the 80–90% range as compared to about 60%, with a maximum around the first order mode resonance frequency of the CMUT membrane. More importantly, the variation of ECR in the whole 1–12 MHz range is small, indicating that ECR can be used as a design guide for a broad range of frequencies. However, assuming that for high power applications like HIFU and ARFI the CMUT will be used around its resonance frequency, in the rest of this paper ECR is evaluated for the case where the output center frequency is around 9 MHz.

## 4. ECR Verification in Small Signal Operation

**Figure 6:**
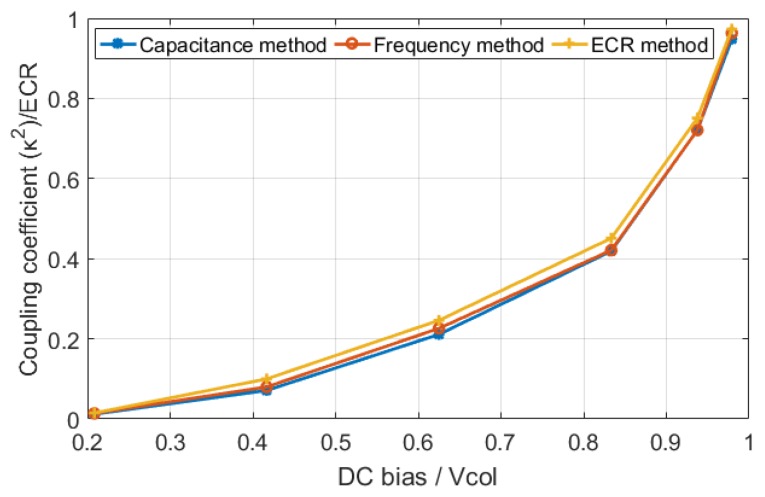
The coupling coefficients of CMUT versus DC bias which show all three methods have the same values in small signal range.

To test the validity of ECR as an equivalent to the coupling coefficient in the small signal regime, the CMUT coupling coefficient is calculated using resonance frequency and capacitance methods and compared to ECR as a function of DC bias. In this calculation, the center element shown in Figure 2 is used with neighboring elements, so that the ECR calculation includes the effects of acoustic crosstalk as well. The resonance frequency method is evaluated by obtaining the electrical impedance of the CMUT in air, and the capacitance is calculated, all using the same model. The results shown in Figure 6 indicate that all three methods follow the same variation in the whole voltage range, and validate that ECR is equivalent to small signal coupling coefficient.

## 5. Large Signal Operation

**Figure 7:**
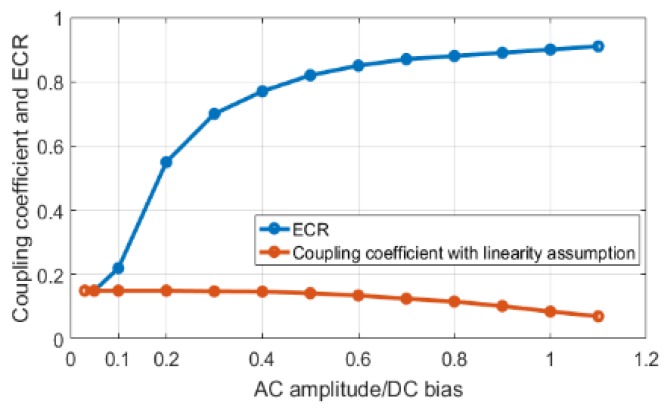
Comparison between ECR and coupling coefficient with linearity assumption over a range of AC signal amplitude to DC bias ratio.

Unlike small signal based coupling coefficient, ECR enables one to explore the large signal energy conversion performance of CMUTs. To demonstrate the need for this approach and determine the boundary between small and large signal operation, the ECR is compared to energy based coupling coefficient calculated with linearity assumption for a range of AC signal values normalized to the DC bias. In this particular case, a DC bias of 0.5 × Vcol is applied along with a 100-cycle AC signal at 9 MHz frequency and the spectrum of the input and output power signals are generated. To calculate the coupling coefficient with linearity assumption, the mechanical energy output only at the input frequency is considered since in a linear system the input and output signals are at the same frequency. This calculation is repeated for increasing AC signal level while keeping the DC bias constant and the results are plotted in Figure 7. As expected, both ECR and coupling coefficient based on linearity assumption have similar values when the AC signal amplitude is below 0.1xDC bias voltage. As the AC to DC voltage ratio increases, CMUT becomes more nonlinear, the vibration frequency, hence the mechanical energy output changes from the input frequency to predominantly to the second harmonic. As discussed in more detail in the Appendix A, for example, with AC only operation, electrostatic forces have mostly DC and second harmonic components, meaning that the coupling coefficient with linearity assumption approaches to 0, whereas ECR increases. This behavior is clearly seen in Figure 7. Therefore, one can conclude that the small signal assumption for CMUTs loses validity after the AC signal level is larger than 0.1 × DC bias for CMUTs. In a way, nonlinearity of the CMUT is not limited to large signal levels, but to the ratio of the pulse/AC signal amplitude as compared to the DC bias. Note that this is the case for transmit operation. In receive mode, the membrane vibration and resulting AC receive signals are much smaller, therefore linearity assumption is satisfied.

**Figure 8:**
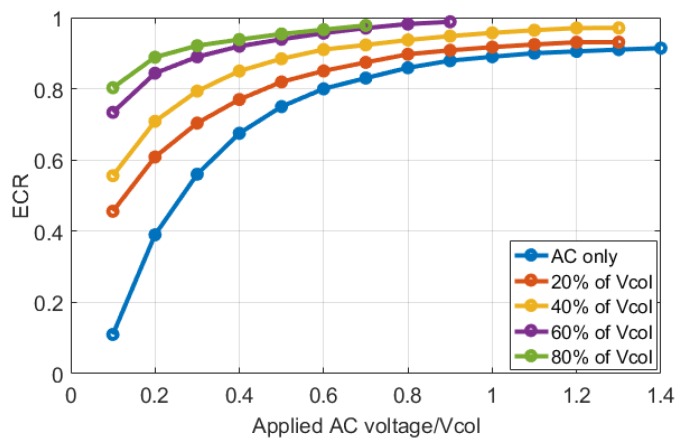
CMUT ECR vs. input signal amplitude.

Consequently, an application of ECR approach is to quantitatively compare CMUT operation for various DC and AC signal combinations. Figure 8 shows the variation of ECR as a function of AC signal amplitude normalized to the collapse voltage for different DC bias conditions. In these curves, high DC bias and low AC signals correspond to small signal operation. The curves are calculated until the instability due to collapse is encountered. In all cases increasing AC signal level increases the ECR with a limit approaching to 1. The interesting observation is that ECR values more than 0.9 can be achieved with AC only (no DC) or small DC bias when large AC signals exceeding the collapse voltage are applied to the CMUT. For example, ECR of 0.9 is obtained by AC only operation with 1.14 × Vcol peak amplitude at 4.5 MHz to obtain pressure output at 9 MHz. Noting that in this case the second harmonic level at 18 MHz is about -26 dB and the maximum pressure is 0.8 dB higher than the DC biased operation, these results show that AC only operation can be quite efficient for large signal CMUT operation [6,10]. As noted elsewhere, AC only operation can mitigate the charging problem as compared to DC biased operation since on average no single polarity bias is applied driving the charges [10]. However, dielectric breakdown still needs to be considered when peak levels of AC signals are large.

### Impact of Parasitic Capacitance on ECR

**Figure 9:**
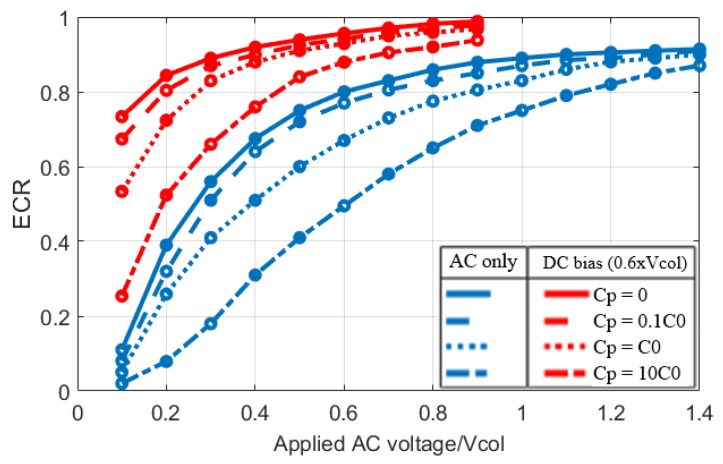
The effect of the parasitic capacitance on ECR in both AC only and DC biased operation.

The impact of parasitic capacitance on ECR is also analyzed as shown Figure 9, where the ECR is evaluated for both DC biased and AC only operation for different parasitic capacitance values. Similar to [4] and [16], the ECR is reduced by parasitic capacitance. This is expected, because parasitic capacitance is a non-moving parasitic capacitor ($\dot{C}\left(t\right)=0$), storing electrical energy without generating mechanical output. Therefore, mechanical energy in the transducer remains the same while total input energy increases, reducing the ECR. It is observed that parasitic capacitance in the order of the active capacitance of the CMUT causes a significant reduction of ECR and it has more impact on AC only operation as compared to DC biased operation. Therefore, implementations of CMUTs that reduce the parasitic capacitance such as fabrication on glass substrate or CMUT-on-CMOS approach would be preferred [17,18,19].

## 6. ECR Application: Mass Loaded and Uniform Membrane Performance Comparison

**Figure 10:**
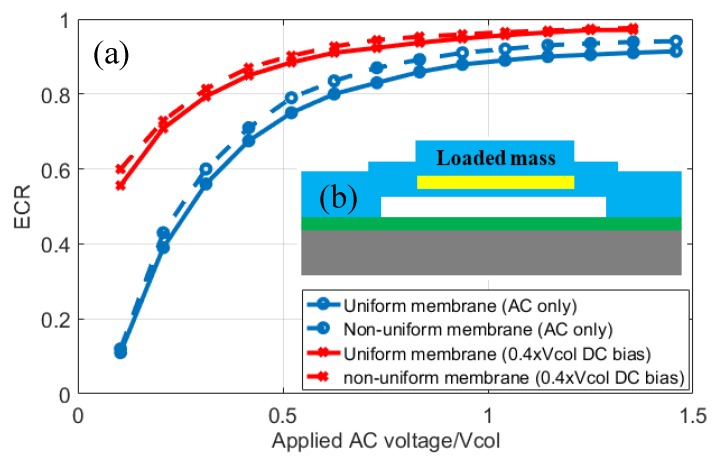
(**a**) The ECR calculation for uniform and non-uniform membranes show non-uniform membrane improves the ECR in both DC biased and AC only operation. (**b**) The schematic of the mass loaded CMUT.

Since the coupling coefficient of both piezoelectric transducers and CMUTs depend on the mechanical boundary conditions [12], a similar dependence is expected for ECR during large signal operation of the CMUT. To demonstrate this possibility to optimize CMUT performance, a uniform membrane CMUT is compared to a CMUT with non-uniform membrane in Figure 10. The uniform membrane device has the parameters shown in Table 2, whereas the non-uniform membrane is designed to have similar lateral size (46 μm membrane pitch) and collapse voltage (32 V). 

Specifically, 1 μm thick silicon nitride is added over the top electrode area to achieve the similar collapse voltage and resonance frequency. The results show that non-uniform membrane improves the ECR for both DC biased and AC only operation as it provides more piston-like motion, with more improvement seen for AC only operation. Given that the ECR formulation allows for arbitrary membrane geometries, this approach can be used for ECR optimization for different electrode size and membrane geometries.

## 7. Experimental Validation of Mechanical Energy Output

**Figure 11:**
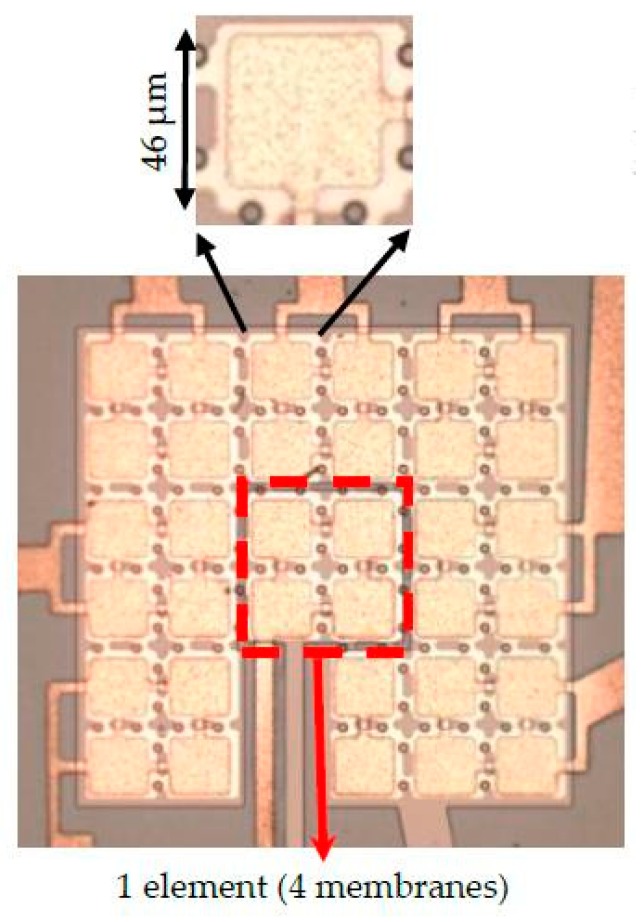
The optical image of the CMUT element that is used in ECR measurements—similar to Figure 2 that used for simulation.

**Figure 12:**
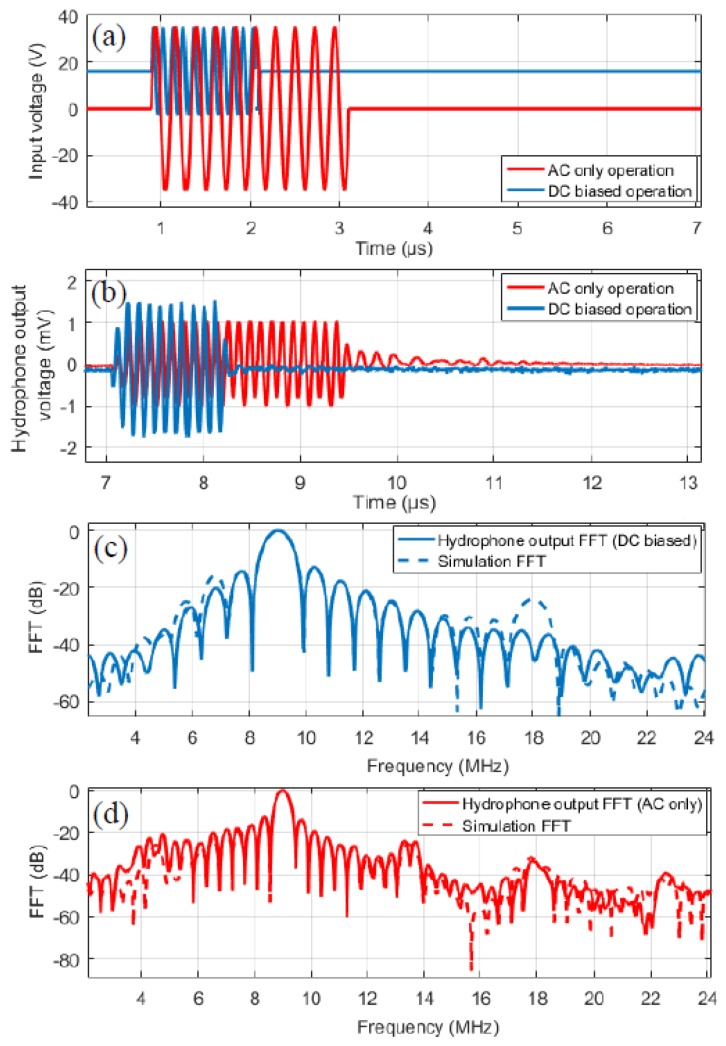
(**a**) Input excitation signal. (**b**) Hydrophone measurement of the CMUT. (**c**) DC biased operation measurement and simulation show the output and input signals are at f_0_ while, (**d**) AC only operation shows the f_0_/2 harmonic is −25 dB lower than f_0_.

**Figure 13:**
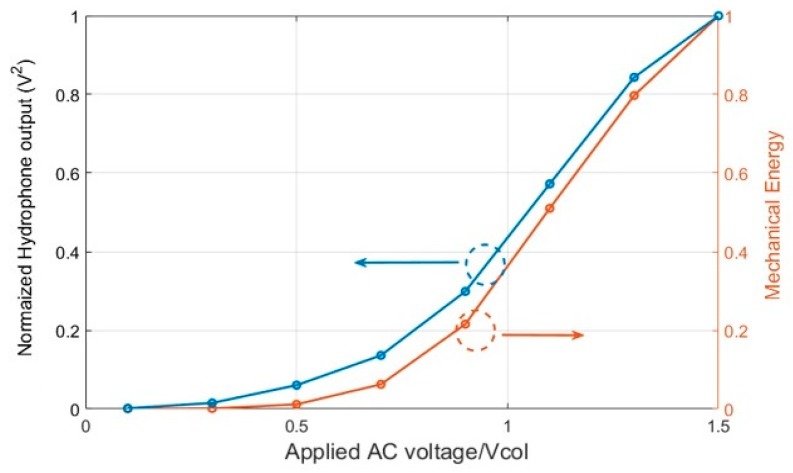
Normalized pressure square, proportional to the acoustic (mechanical) energy output of the CMUT is shown on the left y-axis while the calculated mechanical energy in the CMUT is shown on the right calculation (right y-axis). These variables are plotted as a function of applied AC voltage without DC bias, i.e. AC only operation.

The experimental validation of ECR method is not straightforward as measurement of $\dot{C}\left(t\right)$ is difficult given many sources of parasitic capacitances. Therefore, in order to qualitatively validate the ECR approach, it is considered that the mechanical energy output for a CMUT is proportional to the acoustic intensity, which can be measured through pressure in the far field [20]. For this purpose, a 4 membrane CMUT element is fabricated [21] as part of an array using the parameters of Table 2 as shown in Figure 11. Then, pressure measurements in a water tank are performed and compared with model predictions for both AC only and DC biased operation. The pressure is measured by a hydrophone at a distance of 9 mm. The input signal is a 10-cycle burst with an amplitude of 1.1 × Vcol at f_0_/2 for AC only operation and at f_0_ with 0.6 × Vcol DC bias for DC biased operation demonstrated in Figure 12a. Figure 12 also shows the CMUT hydrophone voltage output results—which were measured by HGL-1000 series hydrophone (ONDA Corp., Sunnyvale, CA, USA) and simulations with large signal model used for ECR calculations. Note that due to frequency doubling, the time domain signal for AC only operation continues twice as long as the DC biased operation (Figure 12a). In both operation modes, simulations agree well with the hydrophone output results in terms of frequency spectrum. To further verify the variation of mechanical energy calculation for ECR, the square of measured output pressure, which is proportional to the acoustic intensity, is qualitatively compared to variation of mechanical energy calculation for full range of AC only excitation voltages. The results shown in Figure 13 show very similar variation where AC peak amplitude is increased up to 1.5 × Vcol, indicating that the mechanical energy obtained by the model is a good indication of actual acoustic output of the CMUT. Overall, these experimental results support the calculations leading to ECR evaluation for CMUT transmit performance in the large input signal range. Note that, although only non-collapsed mode is considered here, the basic behavior of collapsed mode CMUT is the same, i.e., linear time varying capacitor. Therefore, the ECR approach outlined here can be used to evaluate CMUT performance in collapsed more as well. Furthermore, it can be used to devise optimized transmit pulse signals for higher efficiency pulses for imaging applications.

## 8. Conclusions

A large signal CMUT model can be used in conjunction with the energy definition of electromechanical coupling coefficient to determine a parameter called ECR, to quantify efficiency of CMUTs in both small and large signal operation. The model predictions compared with experimental results in the large signal regime show the validity of the model and the mechanical energy calculations for acoustic output of the CMUT. The results indicate that DC bias is not a requirement for efficient large signal CMUT operation. By applying AC only signals at the half of the operating frequency, high power operation with high efficiency (ECR = 0.9) is possible potentially reducing the charging problems. The ECR concept also enables one to evaluate impact of parasitic capacitance and CMUT membrane design on the large signal operation performance.

## Figures and Tables

**Table 1:** Different Definitions of Electromechanical Coupling.

Formula	Notes
$\kappa_{T}{}^{2}=1-{\left(\frac{f_{a}}{f_{r}}\right)}^{2}$	Short circuit resonance frequency $\left(f_{r}\right)$, and open circuit resonance $\left(f_{a}\right)$. [1,2]
$\kappa_{T}{}^{2}=1-\frac{C_{static}}{C_{free}}$	$C_{static}=\frac{Q\left(x\right)}{V};\;x_{DC},\;V_{DC}$ $C_{free}=\frac{dQ\left(x\right)}{dV};\;x_{DC},\;V_{DC}$ [1,2]
$\kappa_{T}{}^{2}=\frac{w_{mech}}{w_{total}}$	$w_{mech}$ is the mechanical work done by the transducer and $w_{total}$ is the total input energy [2].

**Table 2:** CMUT Properties.

Parameter	Value
Membrane size	46 μm × 6 μm
Electrode area	38 μm × 38 μm
Membrane thickness	2.25 μm
Device center frequency	9 MHz
Vacuum gap	95 nm
Dielectric relative permittivity	6.3
Si_x_N_y_ isolation thickness	250 nm
No. of membrane	4
Collapse voltage (Vcol)	32 V
Membrane Poisson ratio	0.22
Membrane Young’s Modulus	110 GPa
Membrane density	2200 kg/m^3^

**Figure A1:**
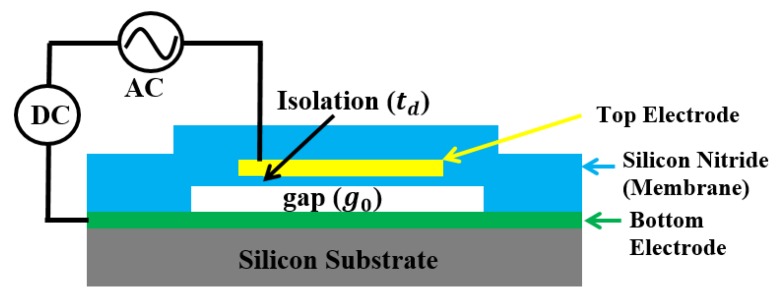
CMUT general schematic and bottom-top electrode location where electrostatic force is applied.

## Appendix A

In order to observe the CMUT operation with or without DC bias, the relationship between input voltage and corresponding force is discussed below. The electrostatic force between bottom and top electrode (defined in Figure A1) caused by voltage applied to CMUT is defined as:

(A1) $$F_{ES}=\frac{\varepsilon_{0}Av{\left(t\right)}^{2}}{2g{\left(t\right)}^{2}}$$

where $\varepsilon_{0}$ is the permittivity of free space (F.m^−1^); κ is the relative permittivity of the dielectric, g(t) is the gap of the CMUT which has the following relation with initial vacuum gap, *g*_0_, and U(t), displacement:

$$g=g_{0}+\frac{t_{d}}{\kappa }+U\left(t\right);\;t_{d}\;is\;isolation\;thickness$$

Usually the CMUT is directly driven with a voltage v(t) that includes DC and AC components (Figure 13), i.e.:

(A2) $$v\left(t\right)=v_{DC}+v_{AC}\cos(\omega t)$$

From (A1) the square of this signal, given in (A2), generates first and second harmonic content depending on the DC bias level and the operation is called DC biased operation:

(A3) $$v{\left(t\right)}^{2}=v_{DC}{}^{2}+v_{AC}{}^{2}cos{\left(\omega t\right)}^{2}+2v_{DC}v_{AC}\cos(\omega t)$$

while in case of no DC voltage applied, the operation mode is called AC only operation and the drive force will include DC and second harmonic components:

(A4) $$v{\left(t\right)}^{2}=\frac{v_{AC}{}^{2}}{2}+\frac{v_{AC}{}^{2}}{2}\cos\left(2\omega \right)t)$$
